# Controlling the screening process of a nanoscaled space charge region by minority carriers

**DOI:** 10.1038/ncomms10108

**Published:** 2016-01-05

**Authors:** Philipp Kloth, Katharina Kaiser, Martin Wenderoth

**Affiliations:** 1 IV. physikalisches Institut, Georg-August-Universität Göttingen, 37077 Göttingen, Germany

## Abstract

The miniaturization of future electronic devices is intimately connected to the ability to control electric fields on the atomic scale. In a nanoscopic system defined by a limited number of charges, the combined dynamics of bound and free charges become important. Here we present a model system based on the electrostatic interaction between a metallic tip of a scanning tunnelling microscope and a GaAs(110) semiconductor surface. The system is driven out of equilibrium by optical excitation, which provides ambipolar free charge carriers, and by an optically induced unipolar tunnel current. This combination enables the active control of the density and spatial distribution of free and bound charge in the space-charge region, that is, modifying the screening processes. Temporal fluctuations of single dopants are modified, meaning we are able to control the noise of the system. It is found that free charge carriers suppress the noise level in field-controlled, nanoscopic systems.

## Abstract (web summary)

Understanding the dynamics of bound and free charges and local electric fields on a nanometre scale are important in scanning tunnelling microscopy and nanoscale electronics. Here, the authors present a model system—a metallic tip near a gallium arsenide surface—for studying such electrostatic interactions.

## Graphical abstract

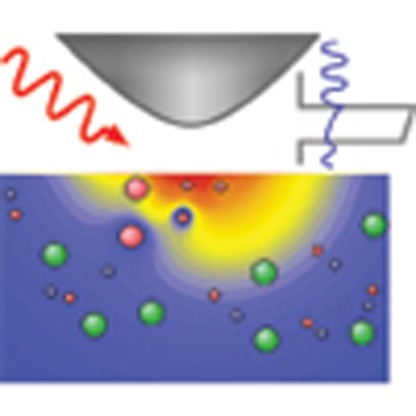

Nowadays, semiconductor-based electronic devices have already reached the nanometre size^1^,^2^,^3^. At this scale, the discreteness of charge, given by, for example, charged single impurities, has already become visible^4^,^5^. The latter are the natural limit of the miniaturization process of future electronic applications. Processes, which define the equilibrium charge state of such a nanoscopic device, are shown in Fig. 1a. A gate voltage ionizes dopant atoms to build up the local space charge region (SCR). A current of free minority charge carriers *I*_H_ towards the field region sets in (Fig. 1a, α). Recombination of carriers inside the SCR results in spatial and temporal fluctuating charges. All this depends on the charge density, the emission (Fig. 1a, β) and capture rate (Fig. 1a, δ) of dopants and the dynamics of the minority charge carriers.

In the following, we describe an approach in which the sharp tip of a scanning tunnelling microscope (STM) serves as the very local gate^4^,^5^,^6^,^7^,^8^,^9^,^10^. Free charge generation by optical excitation and carrier injection by the tunnel current allows driving the system out of equilibrium. We are able to actively control the charge configuration at the surface and thereby establish different screening processes. Values like the carrier flow inside the SCR for different tunnel currents, optical excitation powers and bias voltages can be extracted. Moreover, it is possible to characterize the charge dynamics using a noise analysis of the tunnel current for different charge configurations at the surface.

## Results

### STM on the GaAs(110) surface under optical excitation

**Figure 1:**
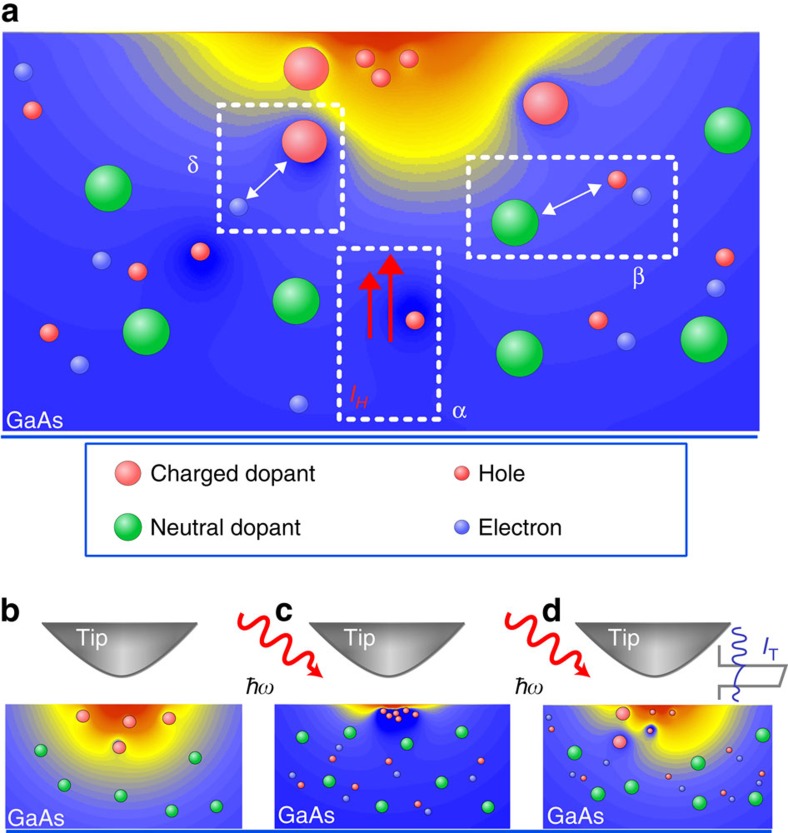
Charge dynamics inside a localized space charge region (SCR). (**a**) The geometry and spatial extend of a SCR is very sensitive to the charge distribution. Besides ionized dopants, a current of minority charge carriers *I*_H_ towards the surface is found (α). This charge replaces the function of the ionized dopants. Doping atoms charge (β) and discharge (δ) by capturing free holes or electrons depending on the hole density at the surface. (**b**) The STM tip-induced potential is used as a local gate electrode to create a SCR of ionized dopants reaching several nanometres into the sample. (**c**) By generating photoexcited electrons and holes, the free charge is separated by the tip-induced field resulting in a hole accumulation at the surface and thereby to a more efficient screening of the potential drop. (**d**) Tunnelling electrons address the hole gas, driving the system out of its equilibrium state. By changing the tunnel current, the hole density and, thus, the screening length of the SCR is controlled.

Positioned only a few Ångstrom above a surface with a low carrier density, the metallic tip induces a nanoscopic SCR inside the sample (Fig. 1b). At an *n*-doped (3 × 10^18^ cm^−3^) GaAs(110) surface with a positive sample voltage of 2.5 V applied, a depleting charge layer is induced extending ∼20 nm into the sample surface with only ∼10 ionized dopants involved^4^.

To get access to the dynamics of this nanoscaled system, we have combined STM with optical excitation (Fig. 1c). Photogenerated electron–hole pairs will be separated by the electric field. In equilibrium, this current of positive minority charge carriers *I*_H_ (*n*-doped) towards the surface is balanced by recombination and thermionic emission. The SCR is modified by the hole accumulation, screening the potential between tip and sample more efficiently. Originally ionized dopants partly discharge, which results in a shift of the surface potential, by default described as a surface photovoltage^11^.

The photogenerated charge accumulation at the surface results in an additional current channel^12^,^13^,^14^,^15^. Carrier injection via the tunnel current *I*_T_ into the valence band distorts the balance of free charge given by optical excitation and locally fixed charge of ionized dopants inside the SCR (Fig. 1d), modifying the non-equilibrium conditions. Surprisingly within the framework of locally resolved surface photovoltage experiments, this carrier injection has been widely neglected up to now^16^,^17^,^18^. The influence of *I*_T_ on the screening process of the SCR has been subject to studies before^13^,^19^,^20^,^21^. Cahill *et al.*^19^ describes this effect as a consequence of a ‘charging' due to high tunnel currents leading to an electron accumulation at the surface and counteracting the photogenerated holes. Similar observations were made by Terada *et al.*^21^ discussed as an increase of hole recombination at increased tunnel currents. Chen *et al.*^20^ describe the change in the SCR as a result of a leakage current modifying the concentration of the free, photogenerated charge and is treated as a parasitic side effect. More sophisticated is the work of Sommerhalter *et al.*^13^. By observing direct tunnelling into minority carriers, they succeeded to develop a model considering the influence of the charge injection by *I*_T_.

In our experiment, *I*_T_ is used as a control parameter for the charge distribution inside the SCR. Unlike previous studies, we keep the tip–sample potential constant when changing the tunnel current. This allows us to study the balance of free and bound charge at the surface by actively tuning the screening process of the SCR and to disentangle and control the dynamics of the system by only changing *I*_T_. At weak photoillumination, we are able to investigate the full range of carrier injection going from low to high currents and thereby at the same time testing and modifying the screening process in three regimes.

### Valence band tunnelling visible in STM topographies

**Figure 2:**
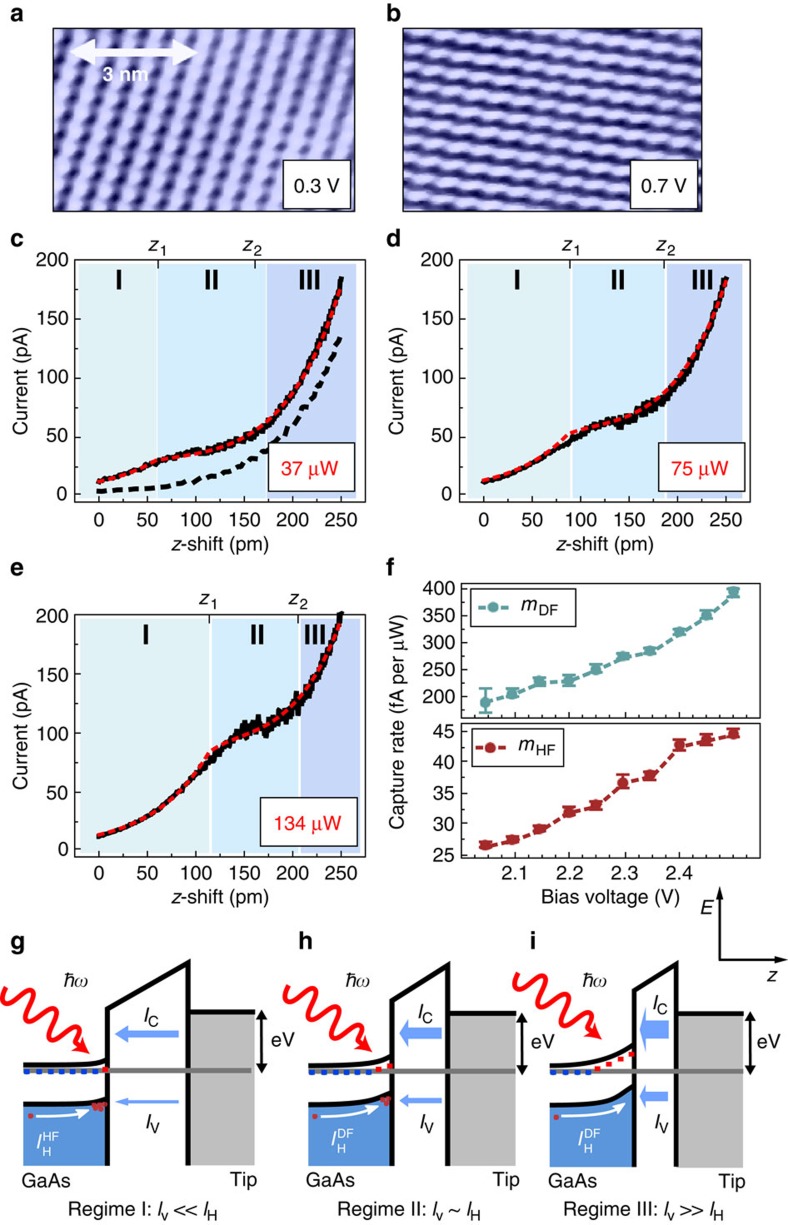
STM topographies, *I*(*z*) spectra and a two-channel tunnelling model. (**a**) The topography at 0.3 V (400 pA) shows the atomic corrugation characteristic for valence band tunnelling. (**b**) At 0.7 V (400 pA), the corrugation originating from conduction band states is visible. (**c**–**e**) Optical excitation-dependent *I*(*z*) spectroscopies (set point: 2.5 V/10 pA). A plateau appears in the curves, which moves in position for increasing optical power. The black-dashed line shows the *I*(*z*) characteristics without optical excitation. (**f**) Hole field-induced *m*_HF_ and dopant field-induced *m*_DF_ capture rate of holes at the surface. The error bars stem from the fitting quality of the model (for details see the Supplementary Notes 4 and 5). (**g**–**i**) A model including both tunnel channels attributes different charge configurations to the three regimes in the *I*(*z*) characteristics. Model calculations are shown as red-dotted lines in **c**–**e**.

In Fig. 2a,b constant current topographies of the optically excited GaAs(110) for positive bias voltages visualize the contribution of tunnelling into photogenerated minority carriers for the first time locally resolved. Different atomic corrugations are observed, which can be attributed to specific resonant surface states positioned energetically inside the valence band (Fig. 2a) and conduction band (Fig. 2b)^22^,^23^ (Supplementary Note 1; Supplementary Fig. 1). The visibility of the valence band corrugation (Fig. 2a) at positive bias voltage is, in contrast to spectroscopic measurements, a direct evidence that tunnelling into photogenerated holes via valence band tunnelling *I*_V_ is possible. Under dark conditions at 0.3 V bias voltage, the conduction band cannot be addressed due to the tip-induced band bending. With optical excitation, it is *a priori* open which tunnel channel (valence or conduction band) is dominant and depends strongly on the density of states and the transmission probability (for details see Supplementary Note 2).

### Accessing different tunneling regimes with *I*(*z*) spectroscopy

Using *I*(*z*) spectroscopy as a function of low optical excitation power *P*_L_ and at fixed bias voltage, we are able to exclusively monitor the influence of *I*_T_ on the screening process inside the surface. The change in the surface potential when decreasing the tip–sample distance by 3 Å is about 5% of the whole SCR and hence is negligible. Figure 2c–e shows a set of *I*(*z*) curves taken at a set point of 2.5 V and 10 pA. It is important to note that this set point allows to directly compare the curves acquired at different *P*_L_ (Supplementary Note 1; Supplementary Fig. 2). A plateau-like signature can be identified in the experimental data, moving in position when changing *P*_L_ and bias voltage (the latter not shown here). In standard tunnel theory, the current dependency *I*(*z*) on the tip–sample distance *z* is described by an exponential relation *I*(*z*)∝exp(−*αz*) with 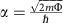 and Φ as the apparent barrier height of the tunnel junction^24^. A closer look on the *I*(*z*) spectra in Fig. 2c–e strongly suggests to divide the curve into three electronic configurations, indicated by the blue shadings in Fig. 2c–e and labelled as I, II and III. In I and III, the *I*(*z*) curves follow the expected exponential dependency. We extract a barrier height Φ for the tunnelling electrons of 2.5 eV (see Supplementary Note 3 and Supplementary Fig. 3 for more details), the same value obtained for the *I*(*z*) characteristics under dark conditions (dotted line in Fig. 2c). Analogous to the topographic analysis, we conclude that conduction band tunnelling is the predominant process at higher bias voltages. Nevertheless, the topography in Fig. 2a demonstrates that the tunnelling into photogenerated holes is possible.

### Modelling the tunnelling into photogenerated minority carriers

To describe the complex *I*(*z*) characteristics under optical excitation, we present a two-channel tunnelling model (Fig. 2g–i), which includes both the current into the valence band *I*_V_ and into the conduction band *I*_C_.

In regime I (Fig. 2e), the valence band tunnelling *I*_V_ is as low that it is not significantly influencing the charge density *n*_H_ at the surface. The electrostatic potential between tip and sample is completely screened by the photogenerated charge, leading to flat band conditions for all optical power ratings *P*_L_. The tunnelling into the valence band *I*_V_ is balanced by a hole current 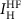. This current can be regarded as a field-driven current induced by the distortion of the hole gas-screening process (hole gas-induced field (HF), see Supplementary Note 4 and Supplementary Fig. 4). In this regime, the transmission probability and not the density of final states of the tunnelling process is the limiting factor for valence band tunnelling *I*_V_. Therefore, *n*_H_ defined by the potential between tip and sample and also the SCR is constant as a function of the tunnel current *I*_T_.

For higher currents, *I*_V_ overcomes 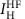 resulting in a filling of final states for tunnelling electrons. Consequently, the hole concentration at the surface decreases (Fig. 2h). At the crossover from I to II (at *z*_1_), valence band tunnelling *I*_V_ and the hole current 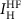 have equalized. In this transition regime II, the hole density *n*_H_ can be actively tuned by adjusting the tunnel current. As this value defines the screening length of the induced field, the controlled decrease of *n*_H_ at the surface leads to an *I*_V_-dependent rebuilding of the SCR (Fig. 2h). As a consequence, two counteracting processes set in. First, due to the rebuilding of the SCR, the conduction band tunnelling *I*_C_ decreases, as a fraction of the bias voltage drops inside the sample. Second, the longer spatial extend of the resulting SCR changes the current of photogenerated holes towards the surface. The holes are accelerated by the tip-induced field, partly screened by ionized dopants, resulting in a more pronounced field-driven hole current 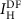 (dopant-induced field (DF), see Supplementary Note 5 and Supplementary Fig. 8). Consequently, *I*_H_ and as a result the valence band tunnelling *I*_V_ increases. Nevertheless, the plateau in the *I*(*z*) curves in II shows that the decrease of the conduction band tunnelling due to the change of the SCR is the dominant effect. At the end of regime II (at *z*_2_), the SCR found without optical excitation is re-established.

Regime III (Fig. 2i) is defined by the lowest hole density at the surface (*n*_H_≈0). The tunnelling into the valence band has even exceeded 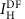. This corresponds to an instant annihilation of all holes participating in the screening process of the potential between tip and sample. The charge is solely provided by ionized, locally fixed dopants. The tunnelling current *I*(*z*) can be described by the sum of the exponential *I*_C_(*z*) characteristics of conduction band tunnelling plus the valence band tunnelling *I*_V_ balancing the field-driven current of holes towards the surface. A further increase of the tunnelling current *I*_T_ does not change *n*_H_ and thereby the SCR. *I*_V_, being limited by 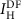, becomes a constant, tunnel current-independent contribution, only dependent on the bias voltage.

To quantify this model, we describe the overall current *I*(*z*) as the sum of valence band *I*_V_(*z*) and conduction band tunnelling *I*_C_(*z*) in the three different current regimes. Our model is able to accurately reproduce the observation (red lines in Fig. 2c–e, Supplementary Note 6). A detailed analysis (Supplementary Notes 4 and 5; Supplementary Figs 5,6,7,9 and 10) shows that both, the hole gas-induced field-driven current 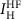 and the dopant-induced field-driven current 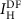, depend linearly on the optical excitation power *P*_L_ (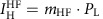 and 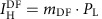). According to the model at *z*_1_ or *z*_2_, the valence band tunnelling has equalized 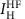 or 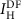, respectively, allowing us to extract the charge generation inside the SCR. The capture rate *m*_DF_(z_2_) is eight to nine times higher in comparison to *m*_HF_ (Fig. 2f). The model allows us to calculate and to separate valence and conduction band tunnelling (Supplementary Note 6; Supplementary Fig. 11). At an optical power of 100 μW, valence band tunnelling contributes 4% at *z*_1_, whereas at *z*_2_ the valence band tunnelling accounts for over 30% of the overall tunnel current. The latter corresponds to an annihilation of 2.5 × 10^8^ holes per second. With a focus diameter of 50 μm and a penetration depth of about 1 μm of the laser light^25^, holes in an excitation volume of (40 nm)^3^ are collected. This volume has the same magnitude as the spatial extends of the SCR under dark conditions, suggesting that in regime III all holes in the tip-induced electric field are depopulated. Both values, *m*_HF_ and *m*_DF_, show a significant bias voltage dependency, which is correlated to the potential drop between STM tip and sample that is needed to be screened (for more details see Supplementary Notes 4 and 5).

To sum up this section, we compare our results and the model to previous studies^13^,^19^,^20^,^21^. Contrary to refs 19, 21, we can exclude the effect of increased electron injection into the conduction band for high tunnel currents leading to a change in the SCR, as the topographic analysis in Fig. 2a,b is able to clearly show additional valence band tunnelling into minority carriers. This mechanism, also discussed in refs 13, 20, reacts very sensitive to the size of the SCR, dependent on the STM tip geometry, the potential between tip and sample and the density of optical excitation. By keeping the bias voltage fixed and applying low optical excitation the *I*(*z*) spectroscopy allows us to develop a quantitative two-channel tunnelling model. An advantage of our experimental conditions in comparison to previous work^13^ is the combination of conduction band *I*_C_ and valence band tunnelling *I*_V_ enabling us to monitor the change of the SCR, having a major influence on *I*_C_, and the behaviour of the tunnelling into photogenerated minority carriers, giving the position of the plateau region in the *I*(*z*) curves, simultaneously. Also, by having a defined SCR in regime I (flat band conditions) and III (TIBB under dark conditions), an extensive computational effort of the tip-induced potential can be avoided.

### Noise analysis of the nanoscaled space charge region

**Figure 3:**
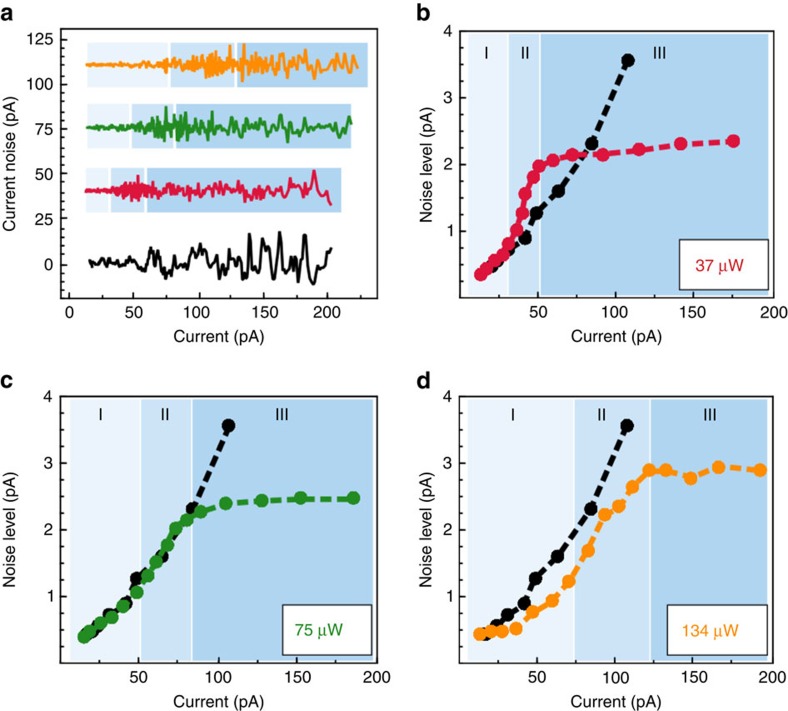
Noise analysis of the *I*(*z*) spectra. (**a**) Current fluctuations of the *I*(*z*) spectra (set point: 2.5 V and 10 pA) plotted against the tunnel current without (black), 37 (red), 75 (green) and 134 μW (yellow) optical excitation. The data sets are displaced vertically. (**b**–**d**) Noise level *σ*_RMS._ plotted against tunnel current.

Concluding, it is obvious that different charge configurations have to be considered in the discussed three regimes. To extract the corresponding dynamic properties, we analyse the noise characteristics of the tunnel current. By subtracting a low-frequency filtered *I*(*z*) curve from the raw data, the signal in a frequency range between 10 Hz and 1.5 kHz is isolated (Fig. 3a). In comparison to the noise without optical excitation, we observe a spontaneous increase in noise in regime II in spectra taken at the photoexcited surface. The standard deviation *σ*_RMS_ in current is plotted against the average current for an optical excitation of 37, 75 and 134 μW compared to the values under dark conditions in Fig. 3b–d. The behaviour of the noise level in the excited case is unequivocally connected to the previously discussed tunnelling regimes, indicated by the blue shadings in Fig. 3b–d.

At 37 μW optical excitation (Fig. 3b), the noise in the tunnel regime I shows the same trend compared to the non-excited case. For regime II, *σ*_RMS_ increases. We attribute this to a non-equilibrium charging and discharging process of dopants^4^,^5^,^9^,^26^ inside the SCR (Fig. 1a,d) due to the carrier injection. By disturbing the hole density at the surface by valence band tunnelling *I*_V_ drives the SCR in metastable configurations. We expect subtle but frequent changes in the geometry of the SCR leading to variations in the tunnelling current, and thereby to additional noise.

In regime III, we observe a saturation of *σ*_RMS_, yielding *σ*_RMS_ (37 μW)<*σ*_RMS_ (0). Previous studies^5^,^27^ have shown that under dark conditions bistable charging processes of doping atoms have to be taken into account. Assuming that these processes define the noise level of the spectra without optical excitation, our results suggest that the presence of free electron–hole pairs suppresses this charge switching in the photoexcited case^9^,^26^. We like to point out that the decrease in noise in comparison to the noise under dark conditions is not the result of a simple decrease of the SCR. Our analysis suggests that in regime III the SCR, normally found under dark conditions, has been rebuilt. Here the charge of the SCR is solely defined by ionized dopants. But even though no more hole accumulation is expected at the surface in the tip-induced field, it is conceivable that the electron–hole pairs, present in the bulk material, cause an additional screening of the SCR. The geometry of the SCR, normally given by the rather stochastic distribution of donors, is smoothed and thereby the charge state of the dopants is stabilized.

By analysing the evolution of the noise level for higher optical power steps at 74 μW (Fig. 3c) and 134 μW (Fig. 3d), this free charge stabilization in the bulk material can actually reduce the noise induced by space charge dynamics even in I and II. In this *z*-range, *σ*_RMS_ decreases monotonically for increasing *P*_L_. In particular for an optical power of 134 μW (Fig. 3d), the noise level is below the values without optical excitation showing that charge switching of bistable dopants is reduced.

From previous studies, the noise level is expected to increase linearly with the tunnel current up to a tunnelling regime close to the point contact of the STM tip^28^. In contrast, in regime III, a saturation in *I*_T_ of *σ*_RMS_ can be observed (Fig. 3b–d), indicating that the resulting *σ*_RMS_ is defined by processes independent of the tunnel current for the optically excited surface. Regarding the dependency on the optical excitation power, we observe a slight increase of *σ*_RMS_ with increasing *P*_L_. Nevertheless, the saturation behaviour for increasing tunnel currents implies that in the frequency range up to 1 kHz charge fluctuations are the dominant contributions to the noise characteristic.

## Discussion

In summary, our results show that tunnelling into minority charge carriers at the surface allows to actively tune the screening process and thereby to decouple the SCR from parameters such as the gate voltage or gate geometry. By controlling the density of minority charge carriers, we are able to suppress the noise in this field determined, nanoscopic system considerably.

For a more detailed analysis of the dynamics, a resolution beyond the kHz bandwidth of the STM is required. This can be achieved using pulsed optical excitation^14^,^21^, which gives the possibility to gain deeper insight into the time scales of the interplay of the tunnel current *I*_T_ and the minority carrier current *I*_H_.

## Methods

### Experimental set-up

The experiments are performed in a custom-built, low-temperature STM working under UHV condition (6 K at a base pressure of *P*<3 × 10^−11^ mbar). For optical excitation, a stabilized continuous wave diode laser at a wavelength of 785 nm and a maximum power of 100 mW is used. The focus diameter of the laser is about 50 μm. With an illumination angle of roughly 30° this results in an optical excited area of 1.57 × 10^−8^ m^2^. The tips are electrochemically etched from a polycrystalline tungsten wire. The typical tip radius is about 10–40 nm. *I*(*V*,*z*) spectroscopy is used to study the effect of the tunnelling process on the photogenerated charge.

## Additional information

**How to cite this article:** Kloth, P. *et al.* Controlling the screening process of a nanoscaled space charge region by minority carriers. *Nat. Commun.* 7:10108 doi: 10.1038/ncomms10108 (2016).

## Supplementary Material

Supplementary Information Supplementary Figures 1-11 and Supplementary Notes 1-6, Supplementary Reference.
