# Supplementary material for: Controlling the screening process of a nanoscaled space charge region by minority carriers
Source: Nat Commun. 2016 Jan 5;7:10108. doi: 10.1038/ncomms10108 (PMC4728339; doi:10.1038/ncomms10108)
Supplement: Supplementary Information — Supplementary Figures 1-11 and Supplementary Notes 1-6, Supplementary Reference. [file ncomms10108-s1.pdf]

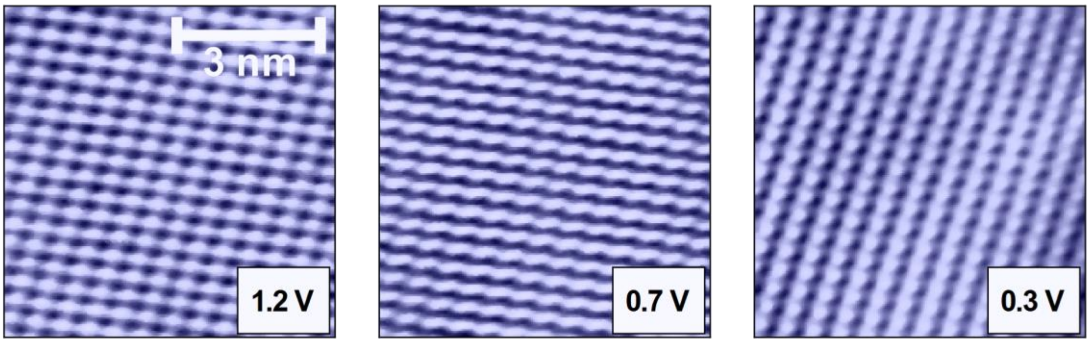

Supplementary Figure 1: Constant current topographies at the optical excited GaAs(110). For higher voltages (0.7 V and 1.2 V) the visible corrugation are characteristic for conduction band tunneling. At 0.3 V one observes the atomic pattern for valence band tunneling.

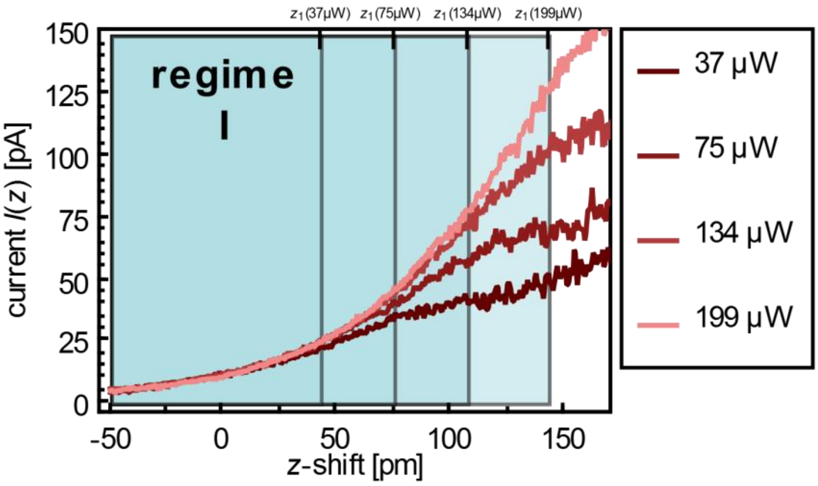

Supplementary Figure 2: *I*(*z*) characteristics at different optical power rates. In the *z*-range of regime I the curves coincide in one master curve (2.5 V and 10 pA). The blue shadings mark the *z*-range of regime I for each excitation power.

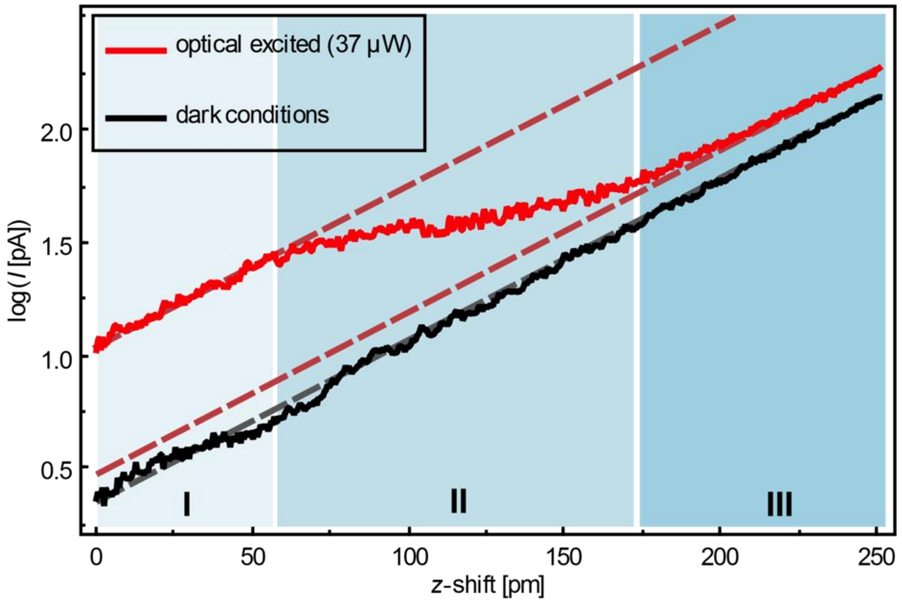

Supplementary Figure 3: *I*(*z*) curves with (red) and without (black) optical excitation plotted on a logarithmic scale. In regime I and II the slope of the curves for the optical excited case is identical to the slope for the data points under dark conditions (set point 2.5 V and 10 pA).

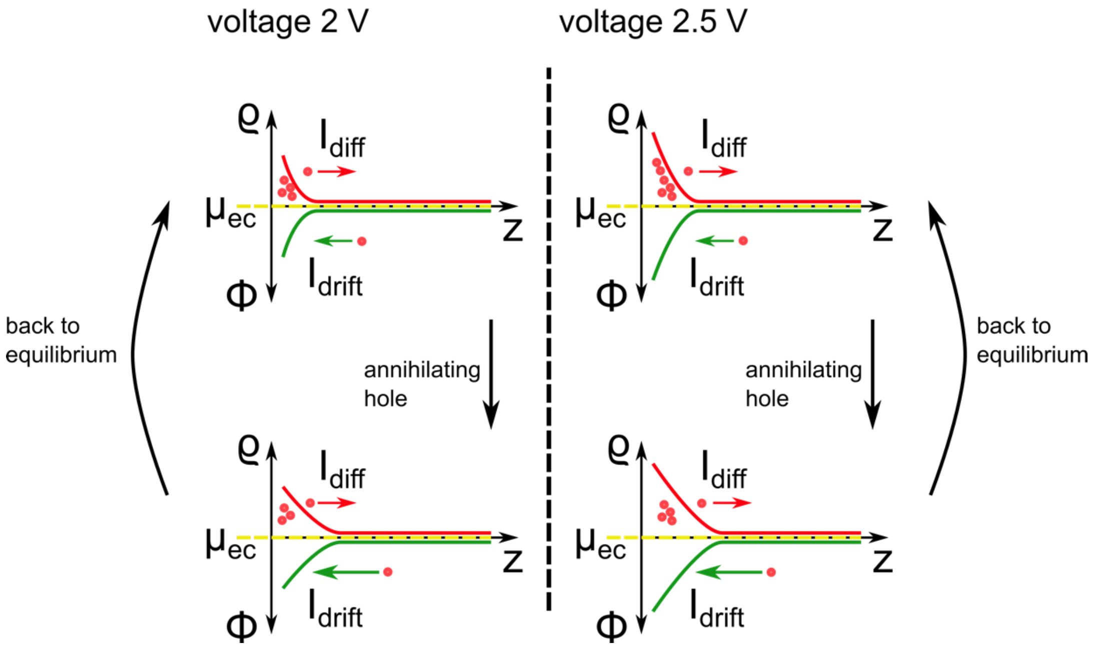

Supplementary Figure 4: Scheme of the charge carrier injection process in regime I. In steady state conditions the hole gas is maintaining balance by a diffusive current I_{diff} and a drift current I_{drift} counteracting each other. This equilibrium is given by the density of holes at the surface, which in return is defined by the potential between tip and sample. Electron injection by the tunneling process distorts the system. Consequently the drift component becomes larger than the diffusional one, driving it back into balance. For the low tunneling currents in regime I this process is faster than the electron injection by I_{V}. Here the potential between tip and sample is solely screened by the photo-generated holes and we have flat band conditions for all tunnel currents.

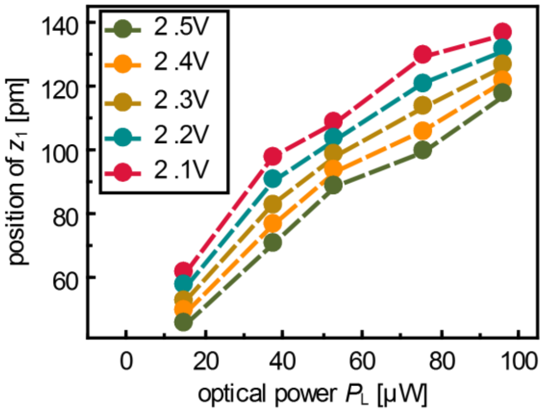

Supplementary Figure 5: z_1 in the $I(z)$ curves plotted against the applied optical power. In a first approximation the data points follow a logarithmic dependency.

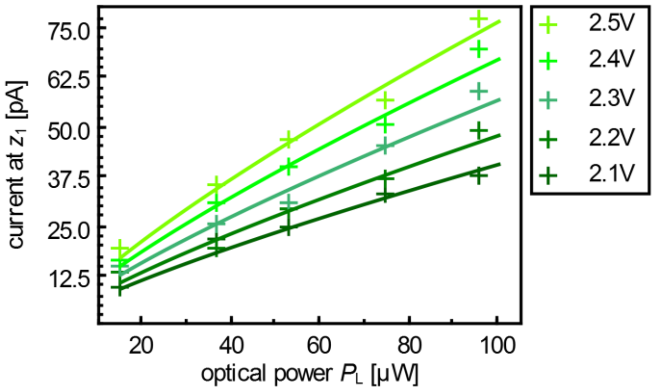

Supplementary Figure 6: $I(z_1)$ plotted against the applied optical excitation power (circles). The solid lines show the result of the fitting to eqn. (8).

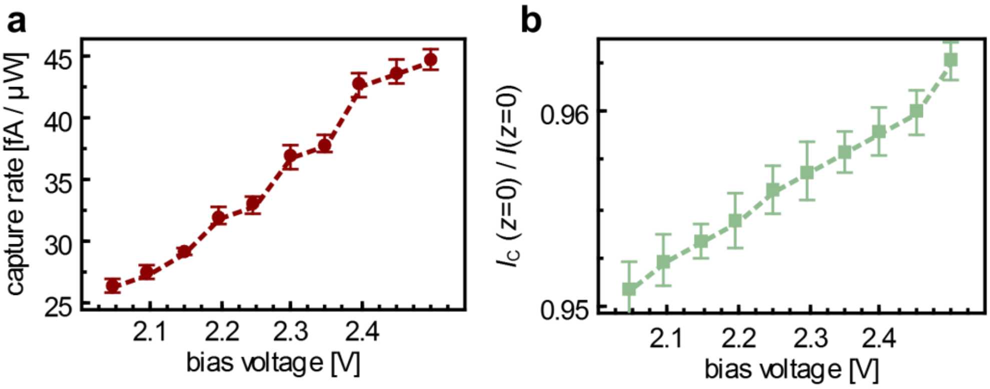

Supplementary Figure 7: Result of the model fit to the data. a Diffusive capture rate of holes $\boldsymbol{m}_{\mathbf{h.F.}}$ plotted against bias voltage. **b** Ratio of conduction band tunneling in relation to the overall tunneling $\boldsymbol{\beta}$ at the set point (z=0). Error bars give the precision of the fitting process.

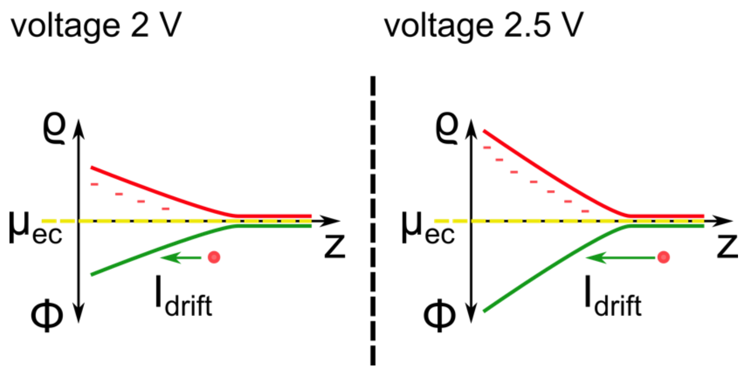

Supplementary Figure 8: Scheme for charge carrier injection in regime II/III. For high tunneling currents the hole gas at the surface is non-existent. The SCR and the electric potential are solely defined by ionized dopants. We expect only a field driven drift current of holes towards the surface I_{drift} which is given by the potential between tip and sample.

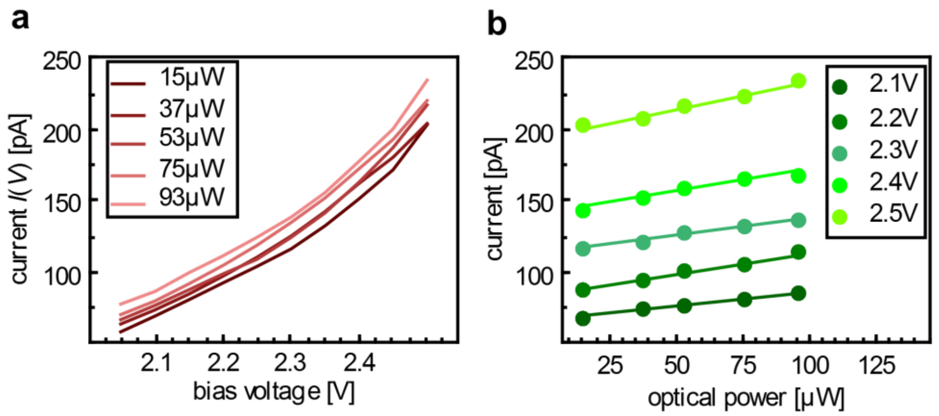

Supplementary Figure 9: Spectroscopic properties in regime III. a $I(V)$ spectra for different optical excitation power ratings in region III. **b** Vertical sections of the $I(V)$ curves shown for different bias voltages. The circles are experimental values. The solid lines a linear fit to these values.

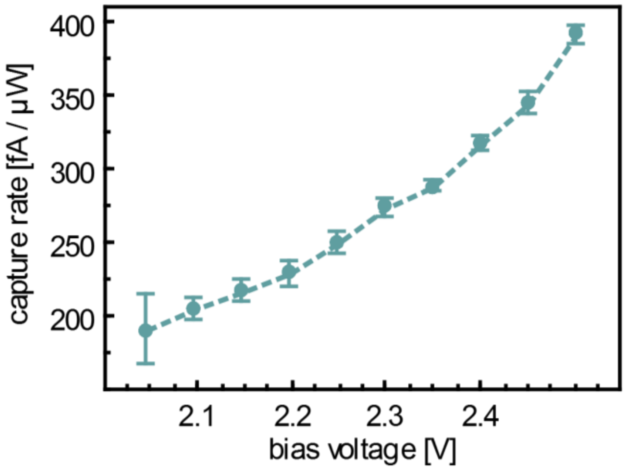

Supplementary Figure 10: Dopant field driven capture rate $m_{D.F.}$ plotted against bias voltage. Error bars show the quality of the fitting procedure.

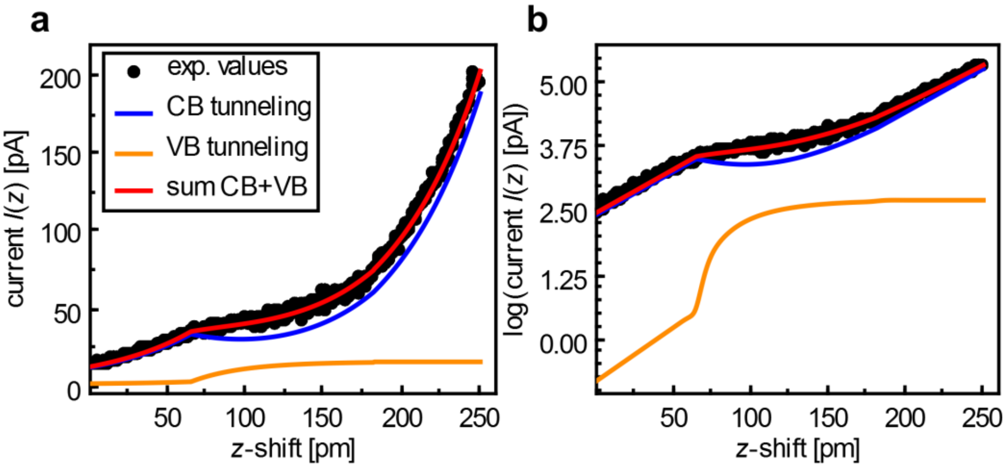

Supplementary Figure 11: Comparison of model and real data. a Model and experimental data for an $I(z)$ spectra taken with 37 µW optical excitation and a set point of 2.5 V and 10 pA. **b** Identical dataset but here plotted on a logarithmic scale. Adjusted parameters: $\Lambda = 0.2$ and $\lambda = 35$ pm.

Supplementary Note 1: Addressing the valence and conduction band surface states at positive bias voltages and the behaviour of the tip-sample distance when changing the laser power P_L

In the main text it is stated that the topographies show the atomic corrugation for valence band states and conduction band states at positive bias voltages. Ebert et al.[1] have shown that it is possible to address three different surface states revealing different atomic corrugations in STM topographies. For conduction band tunnelling one has the transition from a chess-board like pattern for higher voltages (Supplementary Figure 1 at 1.2 V) to a row-like symmetry by decreasing the bias voltage (Supplementary Figure 1 at 0.7 V). For valence band tunnelling at negative bias voltages the atomic pattern shows also a row-like pattern but rotated by 90 degrees. We observe this pattern at low positive bias voltages proofing the possibility to address the photo-generated minority charge carriers (Supplementary Figure 1 at 0.3 V).

In order to ensure the comparability of the $I(z)$ curves for different optical power ratings we have analysed how the tip-sample distance at the set point is influenced by the excitation. This can be best estimated in regime I, i.e. for low tunnel currents. All $I(z,V)$ datasets are recorded at a set point of 2.5 V and 10 pA. The topographies in Supplementary Figure 1 show that for high bias voltages the tunnel current mainly consists of conduction band tunnelling. The model proposes flat band conditions at low currents in regime I. The optical induced additional valence band tunnelling at the set point is defined by the hole density needed to generate these flat band conditions. This density is independent of the applied optical excitation. Therefore we do not expect a change in the net tunnel current I_T when changing the excitation power and therefore identical tip sample distances for each dataset.

This allows us to directly compare the recorded $I(z)$ spectroscopies. Supplementary Figure 2 shows $I(z)$ curves at different excitation densities. In regime I the curves coincide in one master curve.

Supplementary Note 2: Conduction band tunnelling versus Valence band tunneling

In the topographic analysis we observe dominant valence band tunnelling at a bias voltage of 0.3 V. Nevertheless also a current into the conduction band is expected. The determination of the dominant tunnelling channel is a dedicated task. Qualitatively we can estimate this by comparing the density of states and the transmission probability for valence and conduction band tunnelling. Ref.[2] shows the Local Density of States (LDOS) for the GaAs(110) surface energetically resolved. For negative energies for the valence band the LDOS is nearly twice as high in comparison the LDOS for the conduction band. In order to estimate the transmission probability we use an $I(V)$ spectra from Ref.[3]. At 0.3 V bias voltage we have only small tunnel currents. Comparing the amount with current for negative voltages (the energetic position of the photo-generated holes) we expect at least the same or even more current. This rationalizes the observation of mainly valence band tunneling at 0.3 V but also the change of the dominant tunnel channel by going to higher voltages.

Supplementary Note 3: Extraction of apparent barrier height in I(z) spectroscopy

In standard tunnel theory the dependency of the tunnel current on the tip sample distance z is described as:

$$I(z) \propto \exp(2\,\alpha z) \qquad (1)$$

α describes the change in tunnel current when changing z and consists among other values of the apparent barrier height Φ of the tunnel junction, $\alpha = \frac{\sqrt{2\,m\,\phi}}{\hbar}$. Therefore by plotting $I(z)$ spectroscopies on a logarithmic scale (Supplementary Figure 3) the slope of the curves gives directly access to the barrier height Φ.

The trend predicted by eqn. 1 can be nicely seen in case of the curve under dark conditions (Supplementary Figure 3, black curve) showing a clear linear dependency and allows extracting an apparent barrier height of 2.5 eV. This value is characteristic for conduction band tunnelling at the GaAs surface. In the optical excited case the run of the $I(z)$ curve is more complicated. Nevertheless after dividing the curve in the three different tunnelling regimes (Supplementary Figure 3, red curve) one observes an identical slope in regime I and III in comparison to the slope of the curve under dark conditions. This means that in these regimes the apparent barrier height Φ is the same with and without excitation. Consequently in all cases conduction band tunnelling is the predominant process.

Supplementary Note 4: Modelling and extracting the hole current I_{H} in regime I

In the following we give a detailed description on how to model and to extract the hole current I_{H}. Without electron injection by the tunnel current, the density of the hole gas at the surface is defined by the potential between tip and sample (Supplementary Figure 4). Neglecting recombination, there are two currents of minority charge carriers, namely a diffusive component I_{diff} and a drift component I_{drift}. Both components add up to a net current, which is zero under equilibrium conditions. Distorting this balance by electron injection via I_{V}, the drift component increases in order to restore the original state. The net current is then the restoring current, which we call the hole field induced (h.F.) current $I_{\mathrm{H}}^{\mathrm{h.F.}}(P_{\mathrm{L}})$. In regime I this restoring process is faster than the tunnelling rate of I_{V}.
At the position of z_1 we assume that the tunneling into the photo-generated holes $I_{\mathrm{V}}(z)$ has overcome this restoring current towards the surface $I_{\mathrm{H}}^{\mathrm{h.F.}}(P_{\mathrm{L}})$. In regime I we describe the tunnelling into the valence band states with an exponential dependency on the tip height z

$$I_V(z) = I_V^0 \exp(2\,\alpha_2 z) \qquad (2)$$

with $\alpha_2 = \sqrt{2m_e\Phi_V}/\hbar$ with the apparent barrier height Φ_V for valence band tunneling. Then it yields at z_1

$$I_{\mathrm{H}}^{\mathrm{h.F.}}(P_L) = I_V^0 \exp(2\,\alpha_2 z_1) \qquad (3)$$

$$z_1 = \log\frac{I_{\mathrm{H}}^{\mathrm{h.F.}}(P_L)}{I_V^0}/2\alpha_2\ . \qquad (4)$$

In order to analyze the P_{L} dependency of $\mathrm{I}_{\mathrm{H}}^{\mathrm{h.F.}}(\mathrm{P}_{\mathrm{L}})$ we plot z_1 against the optical excitation power (Supplementary Figure 5). In a first approximation $z_1(P_{\mathrm{L}})$ shows a logarithmic behaviour. Taking the exponential dependence of the tunnel current on the distance into account, we describe $I_{\mathrm{H}}^{\mathrm{h.F.}}(\mathrm{P}_{\mathrm{L}})$ with a linear dependency on P_{L}.

$$I_{\mathrm{H}}^{\mathrm{h.F.}}(P_L) = m_{\mathrm{h.F.}} \cdot P_L \qquad (5)$$

To calculate the diffusive capture rate of holes $m_{\mathrm{h.F.}}$ we use the balance of valence band tunnelling I and hole diffusion towards the surface $I_{\mathrm{H}}^{\mathrm{h.F.}}(P_{\mathrm{L}})$ at z_1. The dots in Supplementary Figure 6 show the current at z_1 plotted against the laser power P_{L} for different bias voltages. This current is composed of the tunnelling into the valence band modelled by eqn. (5) and the tunnelling into the conduction band modelled by an exponential dependency on the tip height z

$$I_C(z) = I_C^0 \exp(2\,\alpha_1 z) \qquad (6)$$

with $\alpha_1 = \sqrt{2m_e\Phi_C}/\hbar$ defined by the barrier height Φ_C for conduction band tunneling. The overall current at z_1 can be written as

$$I(z_1) = I_C^0\ \exp(2\,\alpha_1 z_1) + m_{\mathrm{h.F.}} P_L\ . \qquad (7)$$

With the help of eqn. (4) we can transform this into

$$I(z_1) = I_C^0\ \left(\frac{m_{\mathrm{h.F.}} P_L}{I_V^0}\right)^{\frac{\alpha_1}{\alpha_2}} + m_{\mathrm{h.F.}} P_L\ . \qquad (8)$$

It yields

$$I(z=0) = I_C^0 + I_V^0 \qquad (9)$$

so that we can rewrite I_C^0 and I_V^0 as

$$I_C^0 = \beta \cdot I(z=0) \quad \text{and} \quad I_V^0 = (1-\beta) \cdot I(z=0) \qquad (10)$$

Eqn. (8) is dependent on the laser power P_L with the unknown parameter m_{diff}. The ratio of valence and conduction band tunnelling at the set point is modelled by β in eqn. (10). For the tunnel barrier Φ, included in α_1/α_2, the extracted result of 2.5eV/4eV were used (the barrier height for valence band tunnelling is complemented by the additional barrier induced by the band gap of GaAs).
We use a nonlinear fitting routine to adjust eqn. (8) to the experimental tunnel current at z_1. As a side condition of the routine, the parameter β was set to increase monotonically with the bias voltage, as we expect more tunnelling into the conduction band for higher voltages.
The result of the fitting routine can be seen in the solid lines (Supplementary Figure 6). Supplementary Figure 7 shows the adjusted fitting parameters $m_{\mathrm{h.F.}}$ (a) and β (b).

Supplementary Note 5: Modelling and extracting the hole current I_{H} in regime III

In regime III all holes at the surface are instantly annihilated. The SCR and thereby the electric potential at the surface is built up by charged donors (Supplementary Figure 8). In this highly non-equilibrium conditions the diffusive current I_{diff} towards the bulk material can be neglected. As a result the net current of holes $I_{\mathrm{H}}^{\mathrm{D.F.}}(P_{\mathrm{L}})$ is dopant field induced (D.F.) and is solely defined by the field induced drift current I_{drift}.
We assume that the valence band tunnelling $I_{\mathrm{V}}(z)$ has overcome this field driven current $I_{\mathrm{H}}^{\mathrm{field}}(P_{\mathrm{L}})$. We model the valence band tunnelling as a z-independent term

$$I_V(z > z_2) = I_{\mathrm{H}}^{\mathrm{D.F.}}(P_L). \tag{11}$$

Then we can write the overall current in III as:

$$I(z > z_2) = I_C^0 \exp(2\,\alpha_1 z) \cdot \mathrm{TIBB}(z_2) + I_{\mathrm{H}}^{\mathrm{D.F.}}(P_L). \tag{12}$$

TIBB(z) describes the current decrease of conduction band tunnelling because of the SCR restoring. We compare I(V) spectra with different excitation densities taken in the z-range of III (see Supplementary Figure 9a). The identical curvature of the I(V) data points show that these curves only differ in an offset, i.e. shift along the y-axis. As this shift corresponds to the laser induced current into the valence band I_{V}, we can extract $I_{\mathrm{H}}^{\mathrm{D.F.}}$ by plotting the tunnel current I_{T} against the corresponding excitation power P_{L} (see circles in Supplementary Figure 9b) for a given bias voltage. I_{T} follows a linear dependency on the optical excitation power P_{L}. Therefore we model the current of holes towards the surface in this region as the dopant field driven $m_{\mathrm{D.F.}}$ capture rate times the laser power P_{L}

$$I_V(z > z_2) = m_{\mathrm{D.F.}} \cdot P_L. \tag{13}$$

Linear fitting of the data points in Supplementary Figure 9b we extract the field driven rate $m_{\mathrm{D.F.}}$ shown in Supplementary Figure 10.

Supplementary Note 6: Adjusting the model quantitatively to the experimental $I(z)$ curves

This chapter describes the quantitative modelling of the experimental data. In the main article we proposed to describe the recorded $I(z)$ characteristics as the sum of valence band $I_V(z)$ and conduction band tunnelling $I_C(z)$. Then, according to the model for both channels for a given bias voltage, it yields:

$$I_V(z) = \begin{cases} I_V^0 \exp(2\,\alpha_2\, z)\,, & if\ z < z_1 \\ m_{\text{h.F.}} \cdot P_L, & if\ z = z_1 \\ m_{\text{D.F.}}(z) \cdot P_L, & if\ z_1 < z < z_2 \\ m_{\text{D.F.}}(z_2) \cdot P_L, & if\ z > z_2 \end{cases} \qquad (14)$$

$$I_C(z) = \begin{cases} I_C^0 \exp(2\,\alpha_1\, z)\,, & if\ z < z_1 \\ I_C^0 \exp(2\,\alpha_1\, z) \cdot TIBB(z), & if\ z_1 < z < z_2 \\ I_C^0 \exp(2\,\alpha_1\, z) \cdot TIBB(z_2), & if\ z > z_2 \end{cases} \qquad (15)$$

$$I(z) = I_V(z) + I_C(z) \qquad (16)$$

α_1/α_2 are given by the barrier height of the tunneling electrons Φ_1/Φ_2 determined to 2.5 eV/4 eV respectively. The position of z_1/z_2 in the $I(z)$ curves can be directly extracted from the experimental data. The calculation of $m_{\text{h.F.}}$, $m_{\text{D.F.}}(\text{z}_2)$, I_V^0 and I_C^0 is explained previously in the supplemental material.
The parameters $\text{TIBB}(z)$ and $m_{\text{D.F.}}(z)$ are yet undetermined and are strongly related to the restoring process of the SCR at the surface in region II. The model value $\text{TIBB}(z)$ describes the current decrease of the conduction band tunnelling by going from flat band conditions to the tip induced field found at the surface under dark conditions. The additional capture rate introduced by the built up of the SCR is defined by $m_{\text{D.F.}}(z)$. Whereas the limits of $m_{\text{D.F.}}(\text{z})$ are experimentally defined by $m_{\text{h.F.}}(z_1)$ and $m_{\text{D.F.}}(z_2)$, the maximum current decrease $\text{TIBB}(z)$ for the fully restored SCR is adjusted to the experimental data.
We assume that the restoring of the SCR is linearly connected to the applied tunnel current $I(z)$. Then we describe the run of the model parameters $\text{TIBB}(z)$ and $m_{\text{D.F.}}(z)$ with an exponential dependency on the tip height z in the z-range between z_1 and z_2.

$$\text{TIBB}(z) = \Lambda + (1 - \Lambda) \cdot \exp\left(-\frac{1}{\lambda} \cdot (z - z_1)\right) \qquad (17)$$

$$m_{\text{D.F.}}(z) = m_{\text{h.F.}}(z_1) + m_{\text{D.F.}}(z_2) \cdot \left(1 - \exp\left(-\frac{1}{\lambda} \cdot (z - z_1)\right)\right)\,. \qquad (18)$$

Λ defines the percentage of conduction band tunnelling due to TIBB restoring which remains at z_2. λ is given by a fraction of the size in z of regime II. A quantitative comparison of this model with experimental data is shown in Supplementary Figure 11.

Supplementary References

1. Ebert, P. *et al.* Contribution of Surface Resonances to Scanning Tunneling Microscopy Images: (110) Surfaces of III-V Semiconductors. *Phys. Rev. Lett.* **77,** 2997–3000 (1996).

2. Chelikowsky, J. J. R. & Cohen, M. M. L. Self-consistent pseudopotential calculation for the relaxed (110) surface of GaAs. *Phys. Rev. B* **20,** 4150–4159 (1979).

3. Feenstra, R. M. Tunneling spectroscopy of the GaAs(110) surface. *J. Vac. Sci. Technol. B Microelectron. Nanom. Struct.* **5,** 923 (1987).
